# Violence at School and the Well-Being of Teachers. The Importance of Positive Relationships

**DOI:** 10.3389/fpsyg.2019.01807

**Published:** 2019-08-07

**Authors:** Federica De Cordova, Sabrina Berlanda, Monica Pedrazza, Marta Fraizzoli

**Affiliations:** Department of Human Sciences, University of Verona, Verona, Italy

**Keywords:** positive psychology, well-being, teacher, violence at school, social support

## Abstract

In the discipline of positive psychology, “well-being” is considered a fundamental aspect of “human flourishing.” Inherent to this multidimensional model are emotional, psychological, and social forms of well-being, which can be grounded in positive relationships in the work environment. By adopting an interpretive framework that emphasizes these dynamics, researchers are able to focus on elements that actively help sustain the process of flourishing, rather than on negative environmental features that should be avoided if possible. Within this broader discussion, the specific question of teachers’ well-being is one that has global relevance and merits greater academic attention. After all, it has significant consequences for the educational environment and students’ ability to learn. The literature suggests that teachers are increasingly exposed to violence on the part of students and/or their parents. Inappropriate and/or aggressive behavior like this can reduce a teacher’s occupational well-being and make it more difficult to build positive relationships in the classroom. Ultimately, it is one of the most serious work-related stress factors affecting the profession today. Previous studies have attempted to characterize the contexts in which violence occurs, and its negative impact on both the individuals involved and the broader educational climate. Less attention has been devoted to the capacity of teachers to deal with violence and develop a more resilient mindset. The positive psychology perspective focuses on well-being as a multidimensional construct wherein psychosocial and physical comfort does not simply arise provided there is an absence of suffering and violence. Rather, according to this model, such experiences can be counteracted by a capacity to endure and build positive environments. With these considerations in mind, our study presents data gathered in primary and secondary schools in northern Italy. A total 475 teachers completed an online, self-report questionnaire. The results indicate that teachers can experience occupational well-being even if they are subjected to aggressive behaviors. Supportive leadership and good relationships with colleagues may be considered valuable resources for fostering well-being among teachers.

## Introduction

Taking their lead from the field of positive psychology, for nearly 20 years, academics have been working to understand the concept of human well-being in terms of “human flourishing” (Fredrickson and Losada, 2005). This multidimensional model of positive functioning incorporates elements of emotional, psychological and social well-being and seeks to pinpoint those factors that help sustain the process of flourishing, in contrast to approaches that highlight negative environmental features that should be avoided where possible (Seligman and Csikszentmihalyi, 2000). Indeed, “psychology should be able to help document what […] work settings support the greatest satisfaction among workers […] and how our lives can be most worth living. Yet psychologists have scant knowledge of what makes life worth living. They have come to understand quite a bit about how people survive and endure under conditions of adversity. […] This almost exclusive attention to pathology neglects the fulfilled individual and the thriving community. The aim of positive psychology is to begin to catalyze a change in the focus of psychology from preoccupation only with repairing the worst things in life to also building positive qualities” (Seligman and Csikszentmihalyi, 2000, p. 5). This sort of inversion in the theoretical standpoint seems, to us, to offer a particularly fruitful way to approach the issue of well-being among teachers, which has arisen as a common concern around the world, given that it has a bearing on the educational environment and students’ learning skills (Zeinabadi, 2010; Skaalvik and Skaalvik, 2011a,b; McMahon et al., 2014; Benevene et al., 2018; Liu et al., 2018).

Educational contexts are emotionally demanding (Buonomo et al., 2017; Pedrazza et al., 2018). Teachers, in particular, are frequently faced with significant challenges and a lack of adequate resources, both material and immaterial (Hakanen et al., 2006; Bakker and Demerouti, 2017). In such conditions, the teacher-student relationship has a significant bearing on the risk of burnout. In many cultures, teachers are expected both to act as models for their students and to provide them with some form of protection, in a relationship of care. Failure to meet these key duties (Aldrup et al., 2018) is likely to cause stress and harm teachers’ well-being. Recent studies have identified the phenomenon of violence inflicted on teachers in the school setting by students and/or their parents as a serious issue. Indeed, misbehavior and aggression on the part of students can be considered one of the most serious work-related stress factors for the teaching profession, one that is capable of seriously reducing occupational well-being among teachers and preventing them from building positive relationships in the classroom. Violence against teachers at school is therefore an issue with ramifications for societies all over the world, but it remains an under-researched phenomenon. Teacher-directed violence is not defined consistently in the literature (McMahon et al., 2017; Reddy et al., 2018). Existing studies have attempted to characterize the contexts in which violence occurs, and its negative impact on both the individuals involved and the broader educational climate. Less attention has been devoted to the capacity of individuals and groups to deal with violence at school and develop a more resilient mindset, or to describing how such experiences can be endured and counteracted by building a more positive environment. However, if possessing “positive resources” can protect teachers from risk factors and enhance their ability to cope with work-related stressors (Buonomo et al., 2017), it is worth investigating which factors can contribute to and maintain well-being among teachers, even in the face of violence in the school setting.

Looking at the international literature, there are data indicating that 80% of teachers in the United States reported at least one form of victimization at the workplace during the same or previous year in which they completed the research survey (Galand et al., 2007; Espelage et al., 2013; McMahon et al., 2014; Longobardi et al., 2018; Reddy et al., 2018). Although partial, this picture reflects a growth in numbers of aggressive acts directed toward teachers, across different school types and locations and in forms ranging from verbal threats or intimidation to acts of physical and/or sexual violence (Dinkes et al., 2009; Wilson et al., 2011).

Violence is a multidimensional phenomenon produced through various isolated or simultaneous behaviors (Jevtic et al., 2014; Padurariu et al., 2016; Berlanda et al., 2019), which can operate at one or more different levels from the physical to the emotional, psychological and relational. Whereas physical aggression involves the infliction of physical harm, relational aggression is typically characterized by verbal abuse (e.g., shouting and screaming), spreading rumors, and exclusionary behaviors (Lansford et al., 2012). Some researchers have used the terms “bullying” and “victimization” to cover these forms of abuse and violence (Kauppi and Pörhölä, 2012; Anderman et al., 2018); others have applied terms such as “harassment” (Kauppi and Pörhölä, 2009) and “violence against teachers” (Chen and Astor, 2008). Regardless of the exact terminology and categorization of the violence – “student violence,” “school violence,” and “workplace violence,” etc. (Kauppi and Pörhölä, 2012; Skåland, 2016; Anderman et al., 2018) – a picture has emerged in which a significant number of teachers are victimized by their students (Dzuka and Dalbert, 2007; Chen and Astor, 2008; Kauppi and Pörhölä, 2012), with similar features and outcomes all over the world (Van de Vliert et al., 2013; Nielsen and Einarsen, 2018). In the present study, we have chosen the expression “violence directed against teachers” to describe aggressive behaviors perpetrated by students and/or their parents with the intention of harming the teacher. These include harmful verbal and/or physical behaviors (Anderman et al., 2018) and/or damage to personal property (Dzuka and Dalbert, 2007) that violate school rules, negatively affect the school environment, and put the well-being of those involved in the school at risk (Espelage et al., 2013; McMahon et al., 2017).

The well-being of teachers has been widely studied, with research focusing variously on personal, environmental and relational factors (Brouskeli et al., 2018). Aelterman et al. (2007), p. 286) defined teachers’ well-being as “a positive emotional state, which is the result of harmony between the sum of specific environmental factors on the one hand, and the personal needs and expectations of teachers on the other.” Many studies have shown that, in different contexts around the world and regardless of significant differences in education systems (Benevene et al., 2018), violence has a negative impact on the well-being of the teachers affected (McMahon et al., 2014; Aldrup et al., 2018) as well as on the quality of their teaching (Kauppi and Pörhölä, 2012; Montuoro and Mainhard, 2017).

Like violence, well-being is a multidimensional construct (Benevene et al., 2018) that is subject to a range of personal and external factors and that correlates with indicators of resilience (Brouskeli et al., 2018). Occupational well-being among teachers – which is associated with optimal psychological function and their positive experience of work (Ryan and Deci, 2001) – has been defined in terms of both the presence of positive aspects, such as job satisfaction and enthusiasm for work (Benevene et al., 2018), and the absence of negative factors, such as stress caused by difficult relationships with students and their parents (Aldrup et al., 2018). Positive relationships with pupils and parents, and colleagues and school management appear to foster a positive attitude toward the profession (Demirtas, 2010; Berlanda et al., 2017; Buonomo et al., 2017) improve teachers’ level of motivation and engagement with their work, and improve their inclination to apply themselves to developing their professional abilities (Schaufeli et al., 2008; Buonomo et al., 2017).

A positive teacher-student relationship is typically characterized by respect, warmth and trust, as well as low levels of interpersonal conflict (Mansfield et al., 2016; Aldrup et al., 2018). Today, teachers are increasingly dependent on the cooperation of parents and need to maintain a positive relationship with them (Skaalvik and Skaalvik, 2011b). Moreover, establishing positive professional relationships with co-workers and senior members of staff may help in establishing networks of emotional support. Social support from other staff at school can foster well-being and protect against the negative effects of violence at school (Galand et al., 2007; Kauppi and Pörhölä, 2012). Mutually supportive collegial relationships between teachers and school management are useful in developing a working community where problems can be discussed and shared, and potential solutions considered collaboratively (Acton and Glasgow, 2015).

Previous research (Galand et al., 2007) suggests that well-being depends on the presence of positive aspects – such as job satisfaction, good relations with students and their parents and support from school management and colleagues (Independent Variables) – and the absence of negative experiences, such as teacher-directed violence (Dependent Variables). Investigating which factors affect well-being may offer further and deeper insights, which in turn may contribute to the formation of strategies designed to transform possible risk factors into future opportunities to flourish (Brouskeli et al., 2018). While job satisfaction and good relationships can be seen as elements that contribute to well-being at work, many teachers are exposed to violent incidents that impair their well-being. Support from colleagues and school management can mitigate these difficulties and foster well-being.

H1 = We expect a negative correlation between job satisfaction and perceived levels of violence;

H2 = There is a significant, negative correlation between social support and perceived violence;

H3 = Compared to responses from male teachers, responses from female teachers will trend to indicate a greater level of perceived exposure to violence (in terms of the frequency of incidents of perceived violence);

H4 = Longer-serving teachers can be expected to exhibit higher levels of satisfaction than less long-serving colleagues. H5 = It is expected that job satisfaction, social support and length of service have an impact on perceived levels of student-and-parent-perpetrated violence.

## Materials and Methods

An online, self-report questionnaire was administered to a convenience sample of teachers working in primary and secondary schools in north-east Italy. The email addresses used to circulate the questionnaire were provided by the headteachers/directors of the schools involved. Ethical approval for the study was obtained from the Ethics Committee at the Department of Human Sciences at the University of Verona. The questionnaire included a section that explained the nature and the purpose of the study, and a consent form. Written informed consent was obtained from all participants, and participation was entirely voluntary. Participants were informed that they could withdraw from the study or refuse to give information at any time without negative consequences of any sort. We protected the privacy and anonymity of the teachers involved in our research in line with Italian law and the ethical principles of the Italian Psychological Association.

### Participants and Procedure

The study was carried out in March–April 2019. In total, 943 teachers were contacted by email. The research participants were selected on a voluntary basis and a total of 475 questionnaires were completed (response rate of 50.37%). These participants were asked to complete a questionnaire designed to provide measures of job satisfaction, satisfaction with teacher-student and teacher-parent relationships, levels of social support from school management and colleagues, and perceived levels of violence directed against teachers (experienced or witnessed). The large majority of participants were female (345, 72.63%), 127 were male (26.73%), while 3 participants did not indicate a gender (0.64%). The mean age of participants at the time of response was 45.42 years (*SD* = 9.77; *range* = 25–66; 8 missing data, 1.68%), and the mean length of service was 15.21 years (*SD* = 10.75; *range* = 1–43; 2 missing data, 0.42%). Most of the participants were working in upper-secondary school (296, 62.32%), 90 participants were teaching in lower-secondary school (18.95%) and 89 in primary school (18.73%).

### Measures

#### Job Satisfaction

There is little agreement regarding the measurement of job satisfaction (Skaalvik and Skaalvik, 2010, 2013). Locke (1976) defined job satisfaction as a pleasurable or positive emotional state resulting from the appraisal of one’s job (Ho and Au, 2006; Aldrup et al., 2018). As such, we conceptualized teacher job satisfaction in terms of the participants’ affective reaction to their work or to their role as teachers. We therefore designed the study to measure the teachers’ feelings about their work, both in terms of an overall sense of job satisfaction and, more specifically, in relation to a particular aspect of the job, namely the quality of their relationships with students and students’ parents. This area of focus was selected in the belief that positive relationships can contribute to a sense of fulfillment.

*Overall job satisfaction* was measured using a four-item scale (Skaalvik and Skaalvik, 2011a, 2014). The items were: “I enjoy working as a teacher,” “I look forward to going to school every day,” “Working as a teacher is extremely rewarding,” and “When I get up in the morning I look forward to going to work.” Responses were given on a 4-point Likert scale ranging from 1 (complete disagreement) to 4 (complete agreement). Cronbach’s alpha for the scale was 0.823.

*Satisfaction with relationships with students*’ *parents* was measured using a three-item scale (Skaalvik and Skaalvik, 2011b) designed to evaluate the extent to which the teachers felt they were trusted by parents. The items were: “I feel that parents have faith in my teaching,” “Parents are easy to work with,” and “Parents trust and accept my decisions.” Responses were given on a 4-point Likert scale ranging from 1 (complete disagreement) to 4 (complete agreement). Cronbach’s alpha for the scale was 0.859.

*Satisfaction with teacher-student relationships* was measured using a three-item scale (with items similar to those on the previous scale). The items were: “I feel that my students have faith in my teaching,” “My students are easy to work with,” and “My students trust and accept my decisions.” Responses were given on a 4-point Likert scale ranging from 1 (complete disagreement) to 4 (complete agreement). Cronbach’s alpha for the scale was 0.779.

#### Social Support From School Management and Colleagues

To evaluate perceived levels of social support from colleagues and management figures, we used the six-item Workplace Social Support scale from the Job Content Questionnaire (JCQ; Karasek et al., 1998; Hoang et al., 2013) in the Italian version (Baldasseroni et al., 2001). The short version of this scale measures *co-worker support* (3 items, e.g., “My co-workers are friendly”) and *supervisor support* (3 items, e.g., “My supervisor is successful in getting people to work together”). Participants’ responses were recorded on a 4-point Likert scale that ranged from 1 (completely disagree) to 4 (completely agree). Cronbach’s alphas were 0.708 (Co-worker support), and 0.680 (Supervisor support).

#### Violence Toward Teachers

To measure perceived levels of teacher-directed violence, we used the eleven-item scale developed by McMahon et al. (2014). This scale assesses three major forms of violence toward teachers: harassment, property offenses, and physical attacks. Harassment included 5 items (e.g., verbal threats, obscene gestures, and internet victimization). Property offenses included 2 items (theft of property and damage to personal property). Physical attacks included 4 items (e.g., physical attacks and objects thrown). We asked the participants to estimate how often they had been subjected to or witnessed any form of violence directed at a teacher in the 12 months prior to completing the questionnaire, first in relation to incidents perpetrated by *students* and, second, in relation to incidents perpetrated by *students*’ *parents*. Responses were given on a 4-point Likert scale ranging from 1 (never) to 4 (frequently). Cronbach’s alphas were 0.856 (perpetrated by a student), and 0.787 (perpetrated by a parent).

### Data Analysis

Data analysis was performed using IBM SPSS Statistics 21.0. First, for each variable, a composite score was computed by averaging the respective items. Pearson correlation was used to examine the level of association between variables. To test whether different levels of job satisfaction, social support and perceived violence were reported by male vs. female respondents, and less long-serving vs. longer-serving teachers, independent *t*-tests were applied. To explore the potential differences between primary, lower-secondary and upper-secondary teachers, we applied a One-way ANOVA with *post hoc* Tukey. Finally, multiple linear regression analyses were conducted. The regression models included job satisfaction, social support and length of service as predictors, and violence perpetrated by students or students’ parents as dependent variables.

## Results

### Descriptive Statistics and Correlations

Table 1 presents the mean score for the research variables with a note of standard deviations and correlations between variables. In line with our assumptions, the results reveal that more satisfied teachers perceive lower levels of student-and parent-perpetrated violence. These results support our first hypothesis. In regard to social support, the data analysis shows that student-and-parent-perpetrated violence is negatively correlated with perceived levels of support from co-workers, in the first instance, and support from school management in the second. These results support our second hypothesis. There is a positive correlation between teachers’ length of service and their level of satisfaction with their relationships with students. An unexpected result is that the perceived level of support received from colleagues decreases as the length of service increases.

**Table 1:** Descriptive Statistics and Intercorrelations (*N* = 475).

		Mean	*SD*	1	2	3	4	5	6	7
1	Global Job Satisfaction	2.93	0.58	–						
2	Satisfaction with students’ parent-teacher Relationships	3.08	0.59	0.296^***^	–					
3	Satisfaction with student-teacher Relationships	3.17	0.55	0.479^***^	0.464^***^	–				
4	Co-workers Support	2.98	0.69	0.197^***^	0.159^**^	0.141^**^	–			
5	Supervisors Support	2.55	0.70	0.316^***^	0.166^***^	0.201^***^	0.423^***^	–		
6	Violence from Students	1.49	0.42	-0.096^*^	-0.115^*^	-0.127^**^	-0.219^***^	-0.219^***^	–	
7	Violence from Students’ Parent	1.11	0.19	-0.125^**^	-0.222^***^	-0.027	-0.184^***^	-0.184^***^	0.395^***^	–
8	Length of Service	15.21	10.75	-0.087	0.080	0.124^**^	-0.091^*^	-0.036	-0.081	-0.004


*^∗^*p* < 0.05, ^∗∗^*p* < 0.01, ^∗∗∗^*p* < 0.001.*

### Independent *T*-Test and One-Way ANOVA

There were significant differences in the results when broken down by gender or length of service (Table 2). Female teachers reported a higher level of perceived parent-perpetrated violence (*M* = 1.12, *SD* = 0.18) than male teachers (*M* = 1.07, *SD* = 0.18; *p* < 0.020). Female teachers received less support from school management (*M* = 2.50, *SD* = 0.69) than male teachers (*M* = 2.71, *SD* = 0.72; *p* < 0.005). Female teachers derived less satisfaction from their relationships with students (*M* = 3.14, *SD* = 0.55) and students’ parents (*M* = 3.05, *SD* = 0.58) than male teachers (*M* = 3.25, *SD* = 0.53; *p* < 0.050; *M* = 3.18, *SD* = 0.59; *p* < 0.050). Longer-serving teachers may be expected to be better than less long-serving colleagues at managing their classrooms and building good relationships with their students. The results support this, with longer-serving teachers reporting greater levels of satisfaction with student-teacher relationships (*M* = 3.22, *SD* = 0.55) than new teachers (*M* = 3.10, *SD* = 0.55; *p* < 0.020). Moreover, longer-serving teachers reported a lower risk of student-perpetrated violence (*M* = 1.44, *SD* = 0.41) than new teachers (*M* = 1.53, *SD* = 0.43; *p* < 0.050). These results support our third and fourth hypothesis. Less long-serving teachers reported receiving more support from colleagues (*M* = 3.05, *SD* = 0.67) than longer-serving teachers (*M* = 2.89, *SD* = 0.71; *p* < 0.020).

**Table 2:** Differences in the sample means (*N* = 475).

		Men	Women	*t*	Less long-serving	Longer-serving	*t*	Primary school	Lower-secondary School	Upper-secondary school	F
1	Global job satisfaction	2.98 (0.60)	2.91 (0.57)	1.20	2.98 (0.60)	2.88 (0.55)	1.83	2.89 (0.57)	2.93 (0.59)	2.94 (0.58)	0.25
2	Satisfaction with students’ parent-teacher relationships	3.18 (0.59)	3.05 (0.58)	2.19^*^	3.05 (0.60)	3.10 (0.58)	-0.97	2.97 (0.64)	3.09 (0.61)	3.11 (0.57)	1.96
3	Satisfaction with student-teacher relationships	3.25 (0.53)	3.14 (0.55)	2.00^*^	3.10 (0.55)	3.22 (0.55)	-2.42^*^	3.31 (0.51)	3.11 (0.56)	3.14 (0.55)	3.66^*^
4	Co-workers support	3.02 (0.64)	2.96 (0.71)	0.88	3.05 (0.67)	2.89 (0.71)	2.53^*^	3.11 (0.75)	3.10 (0.63)	2.90 (0.68)	5.15^**^
5	Supervisors support	2.71 (0.72)	2.50 (0.69)	2.86^**^	2.61 (0.70)	2.50 (0.72)	1.72	2.60 (0.65)	2.52 (0.68)	2.55 (0.73)	0.27
6	Violence from students	1.48 (0.42)	1.49 (0.42)	-0.02	1.55 (0.43)	1.44 (0.41)	2.33^*^	1.46 (0.38)	1.49 (0.43)	1.49 (0.43)	0.19
7	Violence from students’ parent	1.07 (0.18)	1.12 (0.18)	-2.42^*^	1.11 (0.21)	1.10 (0.16)	0.63	1.19 (0.26)	1.10 (0.16)	1.08 (0.16)	12.96^***^


*^∗^*p* < 0.05, ^∗∗^*p* < 0.01, ^∗∗∗^*p* < 0.001.*

Breaking down the results by school level revealed additional significant differences (Table 2). Primary school teachers are more satisfied with the student-teacher relationships (*M* = 3.31, *SD* = 0.51) than lower-secondary teachers (*M* = 3.11, *SD* = 0.56; *p* < 0.050) and upper-secondary teachers (*M* = 3.14, *SD* = 0.55; *p* < 0.050). Teachers at the primary school level perceived a greater frequency of parent-perpetrated violence (*M* = 1.19, *SD* = 0.26) than teachers in both lower-secondary (*M* = 1.10, *SD* = 0.16; *p* < 0.005) and upper-secondary school (*M* = 1.08, *SD* = 0.16; *p* < 0.001). Furthermore, upper-secondary teachers perceived lower levels of support from colleagues (*M* = 2.90, *SD* = 0.68) than primary (*M* = 3.11, *SD* = 0.75; *p* < 0.050) and lower-secondary teachers (*M* = 3.10, *SD* = 0.63; *p* < 0.050).

### Multiple Regression Analysis of Variables on Violence

Table 3 shows the results of multiple regression analysis. The results indicate that student-perpetrated violence against teachers is negatively associated with perceived levels of support from school management, while parent-perpetrated violence against teachers is positively associated with satisfaction with student-teacher relationships, and negatively associated with both perceived levels of support from school management and the teachers’ level of satisfaction in relation to their relationships with students’ parents. These results support our fifth hypothesis.

**Table 3:** Multiple regression analysis.

Variable	*B*	*SE*	95% CI	β	*t*	*sig.*
Multiple regression analysis of variables on students’ Violence against Teachers (*N* = 475)						
Global Job Satisfaction	0.005	0.039	[-0.080, 0.090]	0.007	0.12	0.902
Satisfaction with students’ parent-teacher Relationships	-0.036	0.037	[-0.115, 0.040]	-0.049	-0.97	0.335
Satisfaction with student-teacher Relationships	-0.047	0.043	[-0.143, 0.044]	-0.060	-1.09	0.277
Co-workers Support	-0.038	0.031	[-0.101, 0.022]	-0.063	-1.25	0.211
Supervisors Support	-0.108	0.031	[-0.182, -0.035]	-0.181	-3.52	0.000
Length of Service	-0.003	0.002	[-0.007, 0.000]	-0.084	-1.82	0.070
Multiple Regression Analysis of Variables on Violence against Teachers perpetrated from Students’ Parents (*N* = 475)						
Global job satisfaction	-0.025	0.017	[-0.068, 0.014]	-0.078	-1.49	0.138
Satisfaction with students’ parent-teacher relationships	-0.077	0.016	[-0.113, -0.041]	-0.242	-4.83	0.000
Satisfaction with student-teacher relationships	0.053	0.019	[0.012, 0.096]	0.154	2.81	0.005
Co-workers support	-0.013	0.013	[-0.040, 0.014]	-0.048	-0.97	0.331
Supervisors support	-0.035	0.013	[-0.063, -0.008]	-0.132	-2.61	0.009
Length of service	0.000	0.001	[-0.002, 0.001]	-0.022	-0.49	0.622


*The 95% bootstrap CIs were computed for unstandardized regression coefficients (1,000 resamples). CI, confidence interval.*

## Discussion

### Resource Identification Initiative

The results of our study show that, in order to improve well-being at work and decrease perceived violence, it is necessary to promote job satisfaction, social support and positive relationships at school. With regard to violence perpetrated against teachers by students, the results show that support from school management operates as a protective factor. Regarding perceived levels of violence perpetrated against teachers by students’ parents, the results indicate that this variable is positively associated with the teachers’ satisfaction with student-teacher relationships, and negatively associated with both the level of support they feel they receive from school management and their level of satisfaction with their relationships with students’ parents. These data are consistent with the existing literature on this topic, according to which (Skaalvik and Skaalvik, 2011b; Mansfield et al., 2016; Benevene et al., 2018), more satisfied teachers are better able to establish positive relationships, resulting in lower levels of perceived violence. A sense of satisfaction with the work environment can foster positive relationships at work, which in turn promotes self-confidence and the development of skills for dealing with aggressive behaviors on the part of students or their parents (Buonomo et al., 2017; Fiorilli et al., 2017).

Breaking down the results by gender: women seem more strongly affected by aggressive behaviors (experienced directly or Witnessed) than their male counterparts. This would appear to be in line with finding -supported by validated data- that, in comparison to men, women tend to perceive themselves as more vulnerable when exposed to threating contexts (Harris and Miller, 2000; Berg and Cornell, 2016), and report a greater number of experiences of aggression. A similar variance emerges in our data in relation to the variable “length of service” (Berg and Cornell, 2016; Reddy et al., 2018), with less long-serving teachers more inclined to feel vulnerable when threatened with aggressive behavior. Interpreting this result is not a straightforward matter, as there are multiple variables that have not been considered in this study. The perception of a lower frequency of violent behaviors among longer-serving teachers might be explained, at least in part, by a greater ability in dealing with potential conflicts with pupils (Reddy et al., 2018). We might also hypothesize that longer-serving teachers have been subject to some sort of selection effect (Berg and Cornell, 2016). One unexpected outcome to emerge in our analysis – again in connection with length of service– is that the perceived level of support from colleagues tends to decrease as length of service increase. When we take into account the school grade, the lowest rate of support by colleagues is highest rate in upper-secondary school. One possible explanation is that longer-serving teachers tend to be more capable at managing problems, which leads their colleagues to offer less support. Whether or not this is the case, it is result that raises some challenging questions and one that merits further, more detailed analysis focusing on contextual variables. Another interesting result is that teachers who perceive more violence on the part of their students’ parents tend to be more satisfied with their relationship with their students but less satisfied with their relationship with the parents. In particular, the primary teachers in our study reported both the highest levels of parent-perpetrated violence and the greatest levels of satisfaction in relation to student-teacher relationships. Having a good relationship with students is usually associated with the perception of reciprocated trust between student and teacher. This may place the teacher, in a sense, in competition with the parents. Furthermore, a teacher who enjoys a positive relationship with a student or students may feel more driven or entitled to make demands on parents in terms of expecting them to cooperate in their child’s education and share the teacher’s mindset, which could be a trigger for confrontation or situations of conflict.

Positive relationships encourage positive emotions and support professional flourishing (Acton and Glasgow, 2015). There is wide recognition in the literature (supported by our own data) that social support has a vital role in teacher’s coping processes (Kauppi and Pörhölä, 2012), because it offers a valuable resource for dealing with stressful experiences. Other studies indicate that colleagues and management figures can offer practical support with problems (Fiorilli et al., 2019) thus fostering emotional well-being and protecting against violent events (Chen and Astor, 2008; Skaalvik and Skaalvik, 2009; Fiorilli et al., 2017, 2019; Aldrup et al., 2018; Anderman et al., 2018; Bounds and Jenkins, 2018; Nielsen and Einarsen, 2018). Moreover, the results of existing studies suggest that higher levels of support from colleagues and school management are negatively associated with exposure to student-and-parent-perpetrated violence (Galand et al., 2007; Reddy et al., 2018). Generally speaking, a school management structure that favors team-working and participative decision-making can help teachers cope with the emotional and psychological activation caused by violent events (Galand et al., 2007; Kauppi and Pörhölä, 2012) and have a demonstrable, direct protective effect on subjective well-being (Reddy et al., 2018). The results of our study highlight the importance of support from head teachers/school directors, which appears to have a direct protective effect that can lead to an overall increase in occupational well-being (Galand et al., 2007; Reddy et al., 2018) and underpin the quality of the educational process (Kauppi and Pörhölä, 2012; Montuoro and Mainhard, 2017).

In spite of initiatives implemented to address the serious issue of violence in schools, it remains an emerging concern. This leads to the conclusion that strategies designed to prevent violence have not yet proven entirely effective, leaving education professionals at risk of some form of aggression. In particular, parents are more involved than ever in the school environment and, despite the importance of positive school-family relationships for the educational process, students and their parents increasingly display a negative attitude toward school staff. However, our data indicate that aggressive behaviors do not always have a profound negative impact on the recipient. Indeed, under certain conditions, professionals can display and leverage personal qualities that help reduce the impact of violence and draw on capacities associated with resilience. These findings can be more clearly understood in terms of the broaden-and-build theory (Fredrickson, 2000, 2001, 2004), which sets negative and positive emotions apart as two separate systems that respond to different psychological needs. While negative emotions are useful in terms of self-preservation, for instance for focusing on potential threats, positive emotions can help in broadening thought–action repertoires and developing personal resources and creative actions. As such, negative and positive emotions work as complementary systems, in which one of the principal functions of positive emotion lies in deactivating the “negative setting” engendered by negative emotion in order to open up resources for resilience (Fredrickson et al., 2000). In these terms, well-being entails the fulfillment of profound psychological needs and should not be understood as the mere absence of negative emotions (Buss, 2000; Ryan and Deci, 2000). Relationships play a crucial role in the process of building resilience (e.g., Le Cornu, 2013; Gu, 2014; Mansfield et al., 2016) and efforts to engender positive emotions by working to develop better relationships should be a keystone of strategies for establishing and sustaining professional flourishing (Acton and Glasgow, 2015).

Our study has certain limitations, which we can acknowledge here. First, it involved a small number of schools, which makes it difficult to draw generalizable conclusions from the data. All the same, our data are in agreement with the existing literature, and we have no evidence that the situation is different in other schools. Second, the cross-sectional nature of our study constitutes another limitation and a longitudinal study would be desirable at this point. Third, we used an online questionnaire, which was issued to teachers in schools selected on a convenience basis rather than through systematic sampling. This choice meant both that teachers who feel less comfortable with web-based data collection were less likely to participate, and that teachers working in schools other than those selected were excluded altogether. Furthermore, the study used a retrospective, self-reporting approach for data collection, which could be affected by recall bias and the subjectivity inherent to self-reported measurements. To minimize these sorts of problems, we used validated questionnaires that have been shown to have good reliability. Finally, to avoid making the research instrument unwieldy, and thus potentially limiting the total number of returned completed questionnaires, we decide to limit the number of the items in the questionnaire (see Phellas et al., 2011). This choice limited the capacity of the study to consider a number of contextual variables, particularly in regard to differences between school environments. As we have suggested already, these would merit further investigation.

## Data Availability

The dataset generated and analyzed for this study is available from the corresponding author upon request.

## Author Contributions

All authors listed have made a substantial, direct and intellectual contribution to the work, and approved it for publication.

## Conflict of Interest Statement

The authors declare that the research was conducted in the absence of any commercial or financial relationships that could be construed as a potential conflict of interest.
